# Tumor-tropic Trojan horses: Using mesenchymal stem cells as cellular nanotheranostics

**DOI:** 10.7150/thno.90187

**Published:** 2024-01-01

**Authors:** Ana Rosu, Behnaz Ghaemi, Jeff W.M. Bulte, Ali Shakeri-Zadeh

**Affiliations:** The Russell H. Morgan Department of Radiology and Radiological Science, Division of MR Research and Cellular Imaging Section and Vascular Biology Program, Institute for Cell Engineering, The Johns Hopkins University School of Medicine, Baltimore, MD, USA.

**Keywords:** Cancer, Nanoparticles, Mesenchymal stem cells, Theranostics, Image-guided therapy

## Abstract

Various classes of nanotheranostics have been developed for enhanced tumor imaging and therapy. However, key limitations for a successful use of nanotheranostics include their targeting specificity with limited off-site tissue accumulation as well as their distribution and prolonged retention throughout the entire tumor. Due to their inherent tumor-tropic properties, the use of mesenchymal stem cells (MSCs) as a “Trojan horse” has recently been proposed to deliver nanotheranostics more effectively. This review discusses the current status of “cellular nanotheranostics” for combined (multimodal) imaging and therapy in preclinical cancer models. Emphasis is placed on the limited knowledge of the signaling pathways and molecular mechanisms of MSC tumor-tropism, and how such information may be exploited to engineer MSCs in order to further improve tumor homing and nanotheranostic delivery using image-guided procedures.

## 1. Introduction

Cancer theranostics can be defined as “see what you treat, and treat what you see” 1. To enable personalized cancer treatment and ease patient care, it is imperative that cancer is first properly imaged and diagnosed before being precisely treated. Combining therapeutic and diagnostic properties within a single theranostic agent is a promising approach: once the theranostic agent is properly localized to allow cancer visualization, therapy can be subsequently performed with minimal side effects.

Theranostic agents can be based on small molecules 2 or nanoparticles (NPs) 3. NP-based theranostics or nanotheranostics are particularly attractive due to the diverse material compositions and physical structures that are available, which allow their use as agents for most cancer imaging or therapeutic modalities 4, 5. In cases where altering the NP composition or structure is not sufficient for imaging or therapy, the necessary diagnostic or therapeutic molecule can be easily adsorbed on, covalently attached to, or carried as cargo within the NPs.

A current key limitation of nanotheranostics is that it is difficult to target, distribute, and retain NPs within the tumor. When administered intravenously (i.v.), NPs marginally accumulate within tumors in a passive fashion due to the enhanced permeability and retention (EPR) effect, with localization limited to vascularized areas 6-8. The mechanism of the EPR effect remains under active debate. While it was initially proposed that inter-endothelial gaps in tumor vasculature were responsible for the transport of NPs into solid tumors, recent studies suggest that up to 97% of NPs enter tumors using active processes through endothelial cells 9. Regardless, targeting NPs through the EPR effect is highly dependent on a multitude of factors, including the type and location of tumors, the degree of tumor vascularization, and the structure of the NPs themselves 10, 11. Even if NPs reach the tumor vasculature, the tumor structure, composed of surrounding blood vessels and a necrotic core, makes it difficult for NPs to penetrate to the tumor center 12. The theranostic use of NPs thus becomes difficult, as visualization and ablation can occur only at the tumor periphery. Further, NP localization within the tumor vasculature is temporary. Due to the high intratumoral (i.t.) pressure, NPs are easily pushed back from the tumor into the circulating blood, clearing out from the tumor within 24 to 72 hours 13. This rapid clearance is an impediment to long-term imaging and therapy of tumors, requiring repeated administration of nanotheranostic agents.

**Figure 1:**
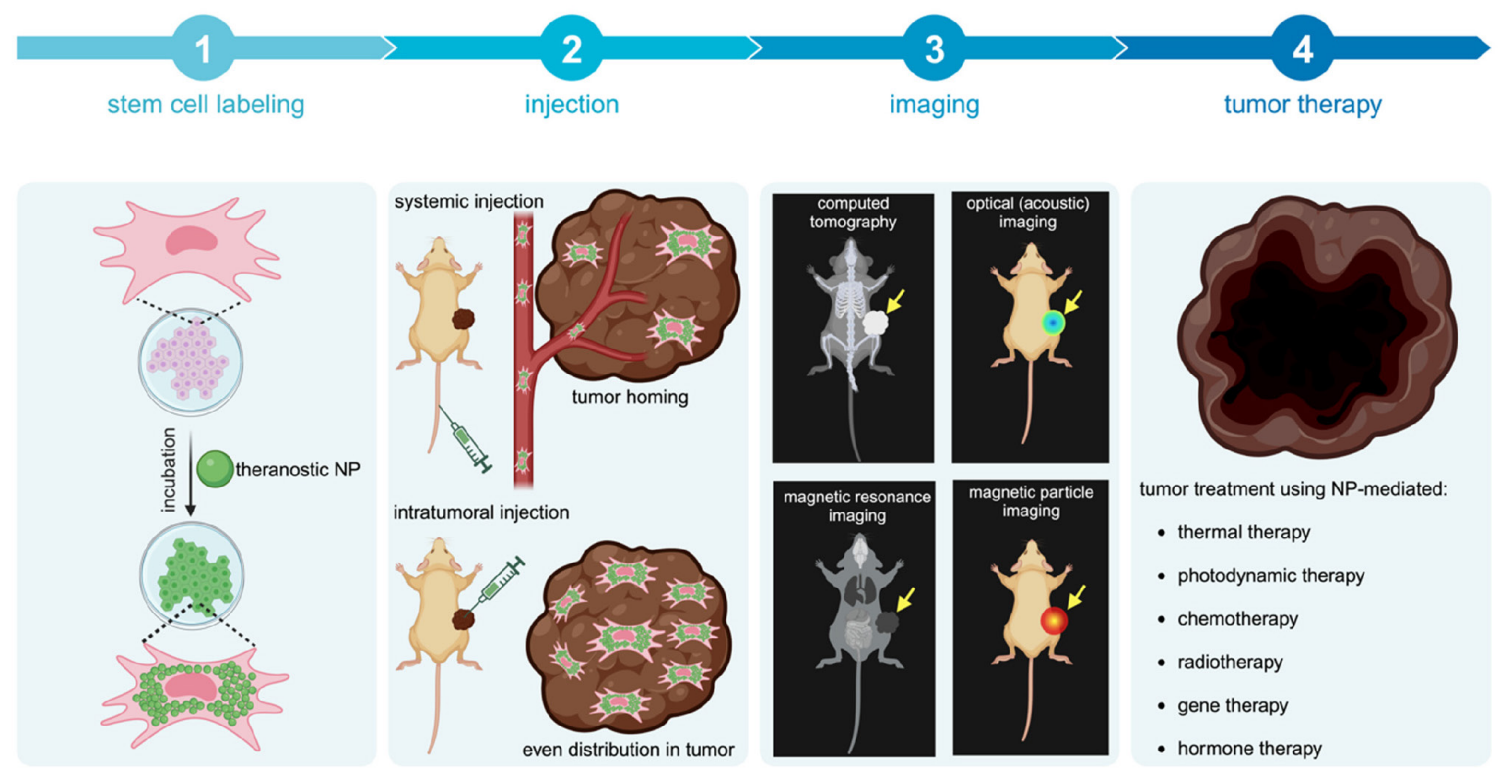
Concept of cellular nanotheranostics following either systemic or intratumoral injection. 1) MSCs are first pre-labeled in vitro with NPs. 2) Labeled MSCs are then injected either systematically or intratumorally. 3) The tumor is imaged using a modality that can detect labeled MSCs in the tumor and off-target sites for making go or no-go decisions on initiating the treatment procedure. 4) When a successful distribution of labeled MSCs is achieved throughout the entire tumor, treatment can be more effective than using “naked” NPs without MSCs.

Potential methods for improving nanotheranostic targeting to tumors include conjugation of targeting moieties to NPs, also referred to as active targeting 14, and cell-mediated delivery of NPs 15, 16. Active targeting generally improves NP localization within tumors, as targeting moieties are designed to bind to receptors overexpressed by cancer cells and can be internalized by appropriate surface receptors. However, tumors are highly heterogeneous in receptor overexpression, making it difficult for NPs with targeting moieties to bind to all cancer cells and/or penetrate to the tumor core 17. Cell-mediated delivery of NPs, using red blood cells, leukocytes, or stem cells, has been explored as an alternative method 15, 16. While red blood cells and leukocytes require surface or genetic modification to target tumors, stem cells are intrinsically tumor-tropic, making them ideal delivery vehicles 18. This review provides an overview of mesenchymal stem cell (MSC)-mediated delivery of nanotheranostics (**Figure 1**), also referred to as cellular nanotheranostics, with the aim of summarizing current progress and defining future directions within the field. Imaging and therapeutic modalities that have been successfully used with cellular nanotheranostics are discussed, with a focus on the potential of MSC-mediated delivery to improve the targeting, distribution, and retention of nanotheranostics within tumors.

### 1.1. Theranostics: An emerging paradigm for imaging and treatment of cancer

Combined cancer diagnosis and therapy, as currently practiced in clinic, has several limitations. Standard imaging modalities such as magnetic resonance imaging (MRI) 3, (single photon emission computed tomography (SPECT), and positron emission tomography (PET) 19 can identify the location and some of the biological characteristics of tumors but remain poorly integrated with the administration of cancer therapies. Chemotherapy and radiotherapy still have limited specificity towards tumors, causing severe toxic side effects and a risk of cancer recurrence 4. Theranostics offers an alternative form of cancer diagnosis and therapy. Imaging can confirm that theranostic agents have localized within tumors prior to initiating therapy, allowing for precise and personalized treatment regimens.

Most advances in theranostics have been in the field of nuclear medicine. However, theranostic radioactive molecules may have toxic side effects due to off-target localization 20 and may fail due to tumor radioresistance 4. Thus, novel precise imaging and therapeutic modalities have been explored for theranostic applications, including fluorescence 21 and photoacoustic imaging (PAI), as well as photothermal therapy (PTT) 22 and photodynamic therapy (PDT) 23. Together with MRI 24, gene therapy 25, and chemotherapy 26, these modalities are discussed more in detail since they can use NPs as theranostic or carrier agents.

### 1.2. Theranostic NPs used with MSC-mediated delivery

The composition of cancer nanotheranostics is either an NP containing a single material that has both imaging and therapeutic properties, or NPs composed of an imaging agent and a therapeutic agent 4. For the former category, gold and magnetic metal oxide NPs have been widely used for imaging and therapy. Gold NPs (AuNPs), convert absorbed light to ultrasound waves for PAI and to heat energy for PTT 27-29. Magnetic metal oxide NPs can serve as negative contrast agents for T2-weighted MRI 30 and magnetic particle imaging (MPI) 31 or, when exposed to intratumoral oxidation and an acidic environment, can release metal ions that serve as T1-weighted contrast agents 32. If exposed to alternating magnetic fields, magnetic metal oxide NPs can also be used for hyperthermia, where they heat up and ablate surrounding cancer tissue 33, 34. One particularly common species of theranostic NPs are quantum dots (QDs), used for PAI or fluorescence imaging, coated with photosensitizing molecules, which can be employed for PTT or PDT 35. Alternatively, a theranostic NP can be enhanced by complexation with genes or chemotherapeutic agents 24.

### 1.3. Signaling pathways and molecular mechanisms of MSC tumor-tropism

MSCs are multipotent stem cells that can differentiate into osteocytes, chondrocytes, adipocytes, and other lineages 36. The primary sources of MSCs for clinical applications are bone marrow, umbilical cord blood, and adipose tissue. Generally, MSCs express the markers CD90, CD73, and CD105, but the surface marker profiles of MSCs derived from different sources tend to vary 37. Depending on their source, MSCs may have different proliferation and downstream cell differentiation rates.

MSCs are recognized for their immunomodulatory properties, making them attractive therapeutic candidates for various clinical applications. They offer several benefits over other stem cell types. Compared to induced pluripotent stem cells (iPSCs), MSCs are more cost-effective and readily available as an off-the-shelf product 38. Furthermore, MSCs have demonstrated tumor-tropism towards a diverse range of cancer types, unlike neural stem cells (NSCs) that have been primarily used for glioma treatment 39. Tumor-tropism, a process by which MSCs selectively migrate towards tumor sites, involves a complex and not fully understood interplay of signaling pathways mediated by various cytokines, chemokines, and growth factors (**Table 1**). Different cancer types express unique cytokine profiles, potentially rendering one cytokine more influential for MSC tumor-tropism in a specific cancer type than in another. Additionally, MSCs derived from varying sources may exhibit different responses to certain cytokines, a factor that has not been fully considered in previous studies.

The most studied signaling molecules involved in MSC tumor-tropism are tumour necrosis factor-α (TNF-α), interleukin (IL)-6, and stromal cell-derived factor-1 (SDF-1). After systematic administration, MSCs have been found to home towards tumors via TNF-α signaling 40. TNF-α induces vascular cell adhesion molecule-1 (VCAM-1) and the α1β1 integrin or very late antigen-1 (VLA-1) expression on MSCs through the phosphoinositide 3-kinases (PI3Ks)- and nuclear factor kappa B (NF-κB) pathways, which then enables MSCs to adhere to endothelial cells in the tumor vasculature 41. IL-6 secreted by tumors has been found to bind to IL-6 receptors and glycoprotein 130 (GP130) on MSCs, inducing chemokine (C-X-C motif) ligand 7 (CXCL7) expression and the signal transducer and activator of transcription 3 (STAT3) pathway activation which have respective roles in cytokine feedback loops and MSC migration 44.

CXCL7 from MSCs interacts with cancer cells via the IL-8 receptor, also known as C-X-C chemokine receptor type 2 (CXCR2), causing tumors to further synthesize IL-6 and IL-8 (another cytokine involved in tumor-tropism). Activation of the STAT3 pathway induces the NF-κB-IL-6-STAT3 cascade, leading to modest extracellular signal-regulated kinase (ERK) activation and longitudinal cytoskeletal organization in MSCs, controlling movement. Tumor-secreted SDF-1 also induces cytokine positive feedback loops, binding to C-X-C chemokine receptor type 4 (CXCR4) on MSCs and causing MSCs to secrete SDF-1 which acts in an autocrine manner 50. SDF-1 binding to CXCR4 on MSCs activates Janus kinase 2 (JAK2)/STAT3 and ERK/ mitogen-activated protein kinase (MAPK) signaling, which further induces focal adhesion kinase (FAK) and paxillin signaling that causes cytoskeletal reorganization characteristic of migratory phenotypes 51. Several other cytokines, chemokines, and growth factors have been identified to play roles in MSC tumor-tropism, including IL-8, CXCL1, monocyte chemoattractant protein-1 (MCP-1), transforming growth factor beta (TGF-β), platelet-derived growth factor (PDGF), hypoxia-inducible factor (HIF)-1α, placental growth factor (PGF), CXCL16, and colony stimulating factor (CSF)-1. However, more studies are needed to reveal the exact signaling pathways that promote cell migration, and, perhaps more importantly, how they interact with TNF-α, IL-6, and SDF-1. It is interesting to note that IL-6 and HIF-1, both associated with tumor hypoxia, have been shown to enhance MSC tumor-tropism 45. Traditionally, highly hypoxic tumors are resistant to immunotherapy 63, chemotherapy, and radiotherapy 64. The capacity of MSCs to migrate towards hypoxic tumors suggests that MSC-mediated delivery of therapeutics could address this gap in current therapies, treating tumors that would otherwise be unresponsive.

### 1.4. Significance of MSC-mediated delivery of nanotheranostics

**Figure 2:**
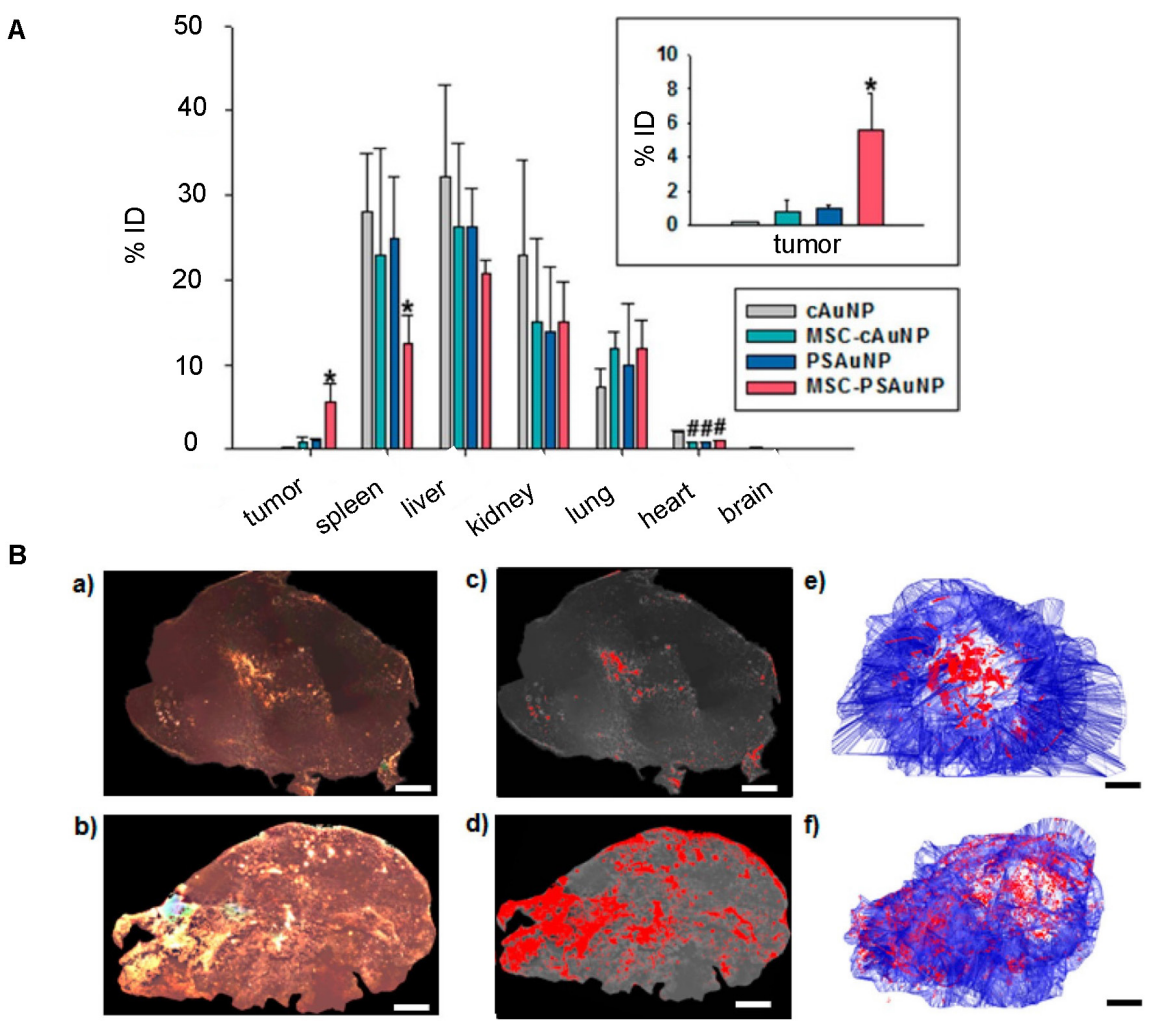
** (A)** Biodistribution of i.v. injected “naked” AuNPs and AuNP-labeled MSCs, in which AuNP-labeled MSCs achieved 5.7-fold higher AuNP delivery in the tumor compared to naked AuNPs. cAuNP and PSAuNP stand for control AuNPs and pH-sensitive AuNPs, respectively. Adapted with permission from 67, copyright 2015 ACS Publications. **(B)** Distribution of AuNPs post-i.t. injection, where AuNP-labeled NSCs (b, d, f) homogenously delivered AuNPs (dense bright signals) throughout the tumor, in contrast to naked AuNPs that are mostly cleared with some remaining in the center of injection (a, c, e). Adapted from 68, courtesy of ACS Publications.

For nanotheranostics to reach full potential, it is essential that theranostic NPs accumulate, distribute, and remain within tumors to allow comprehensive imaging and precise therapy. The inherent tumor-tropism of stem cells has enabled improved NP delivery. For accumulation, there is extensive evidence from MRI cell tracking that iron and gadolinium-based NP-labeled MSCs ultimately home towards tumors 65. Further, MSCs loaded with therapeutic NPs composed of paclitaxel (PTX) and poly (lactic-co-glycolic acid) (PLGA) have been found to localize in prostate, lung, and glioma tumors post-i.v. injection and increase survival rates 66. The success of MSC-mediated NP delivery extends to theranostic NPs. As an example, Kang et al. achieved a 5.7-fold higher AuNP delivery to tumors for i.v. injected AuNP-labeled MSCs 67 compared to “naked” AuNPs (**Figure 2A**).

**Figure 3:**
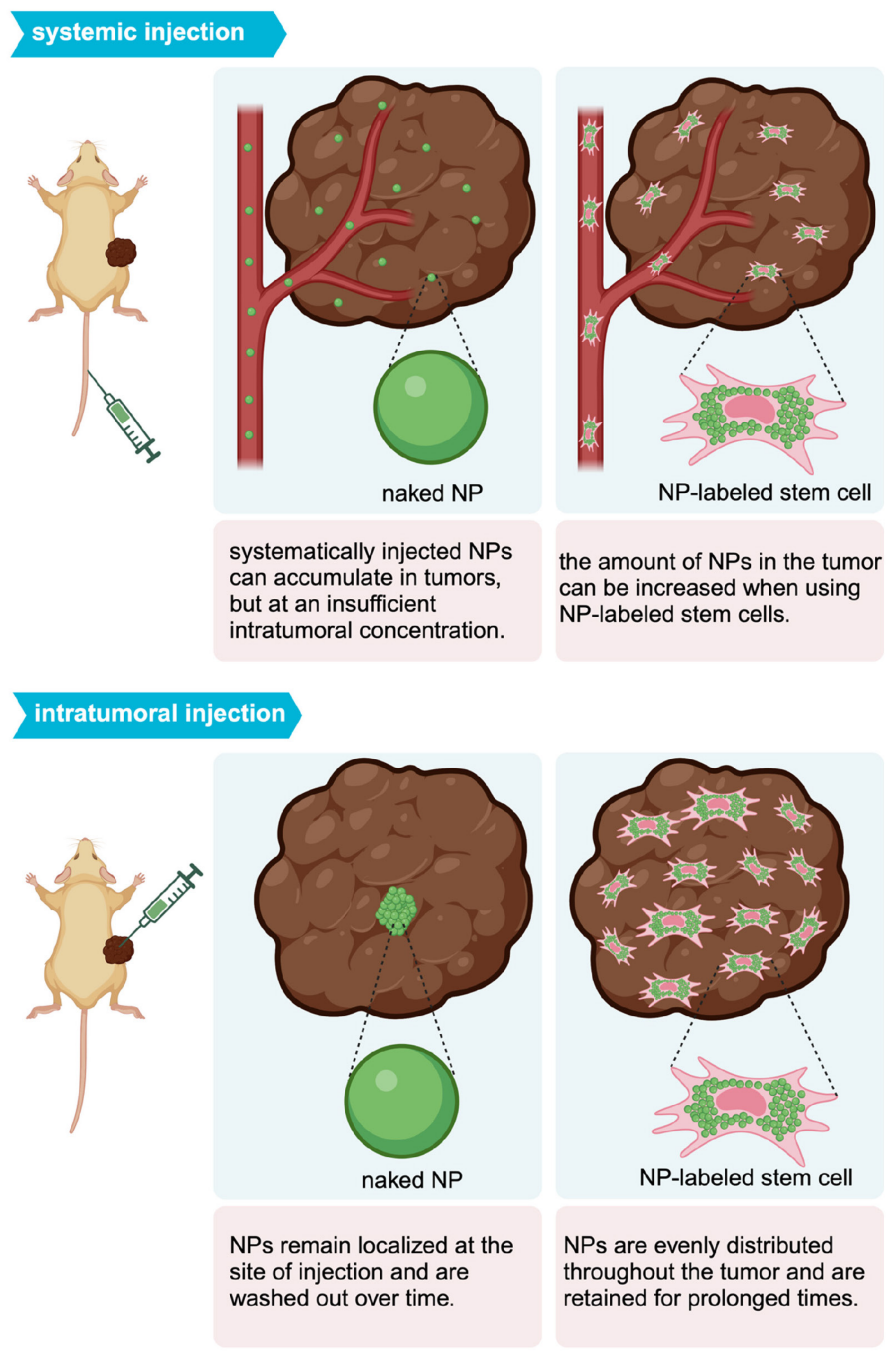
Intratumoral distribution profile of theranostic NPs using different delivery routes.

Mooney et al. were able to homogeneously distribute AuNPs throughout a glioblastoma after i.t. injection of AuNP-labeled NSCs (**Figure 2B**) 68. With PAI, Xu et al. 69 and Huang et al. 70 were also able to demonstrate tumor areas with increased signal after MSC-mediated NP delivery. **Figure 3** illustrates the expected distribution profile of theranostic NPs within the tumor following i.t. or systemic delivery for naked NPs or NPs loaded in MSCs.

**Figure 4:**
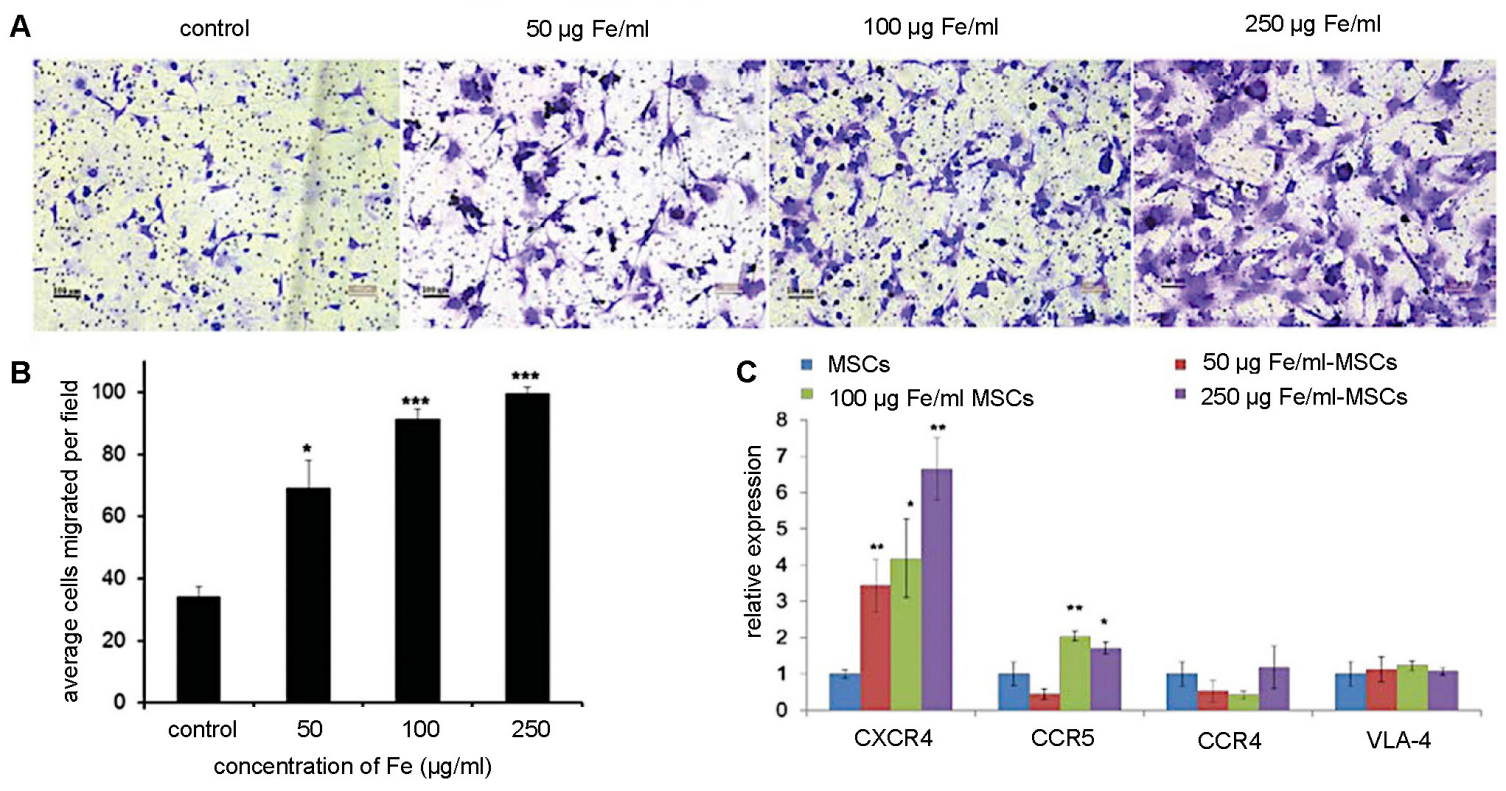
**(A)** Trans-well pictures and **(B)** quantitative analysis of migrating MSCs labeled with various concentrations of magnetic nanoparticles (MNPs). MSCs labeled with increasing concentrations of MNPs have enhanced cell migration towards cancer cells. **(C)** Real-time PCR shows increased CXCR4 expression correlating to higher MNP concentrations used for labeling. Adapted from 73, courtesy of WILEY.

Although retention time varies with tumor type, MSC-mediated NP delivery in general prolongs NP retention compared to naked NPs. Through the EPR effect, naked NPs accumulate within and clear from tumors between 24 and 72 hours post-administration 13. On the other hand, Layek et al. found that Cy5.5-labeled MSCs injected i.v. remained within ovarian and lung tumors for 28 and 10 days, respectively 71. Kim et al. indicated that MSCs labeled with gadolinium-chelate NPs can be detected *in vivo* for at least 21 days, using a colorectal cancer model 72. In both these studies, MSCs homed towards tumors within 24 hours of administration. As for SPIO labeling, multiple studies have demonstrated that iron uptake upregulates CXCR4 expression on the surface of hMSCs (**Figure 4**), providing a potential explanation for the prolonged retention of (SPIO-based) NP-labeled hMSCs within tumor sites 69, 73, 74.

## 2. Imaging modalities for cellular nanotheranostics

Imaging modalities used for cellular nanotheranostics include MRI 75, PAI 76, and fluorescence imaging 77, all of which can use NPs as imaging agents. MRI is the only modality for which NPs have been clinically approved as imaging agents: superparamagnetic iron oxide (SPIO) NPs have been used for diagnosis of liver cancer 78 and lymph node metastases 79, 80. Only one clinical study has been performed using SPIO-labeled MSCs in patients with neurodegenerative disease 81. Small molecule Gd(III) complexes are commonly used as MRI contrast agents in the clinic, but Gd(III)-doped NPs are unlikely to be ever approved due to their slow body clearance with potential toxicity concerns.

While NPs are neither used in the clinic for PAI and fluorescence imaging, they have shown clear potential as imaging agents in pre-clinical animal studies 82-85. AuNPs are preferred for PAI due to their high and stable signal and size- and shape-tuneable absorption rates 86. Fluorescence imaging has been performed with a wide range of NPs that are either inherently fluorescent or loaded with fluorophores, including semiconducting polymer NPs and inorganic NP-based fluorescent probes such as QDs 77, 87.

### 2.1. MRI

**Figure 5:**
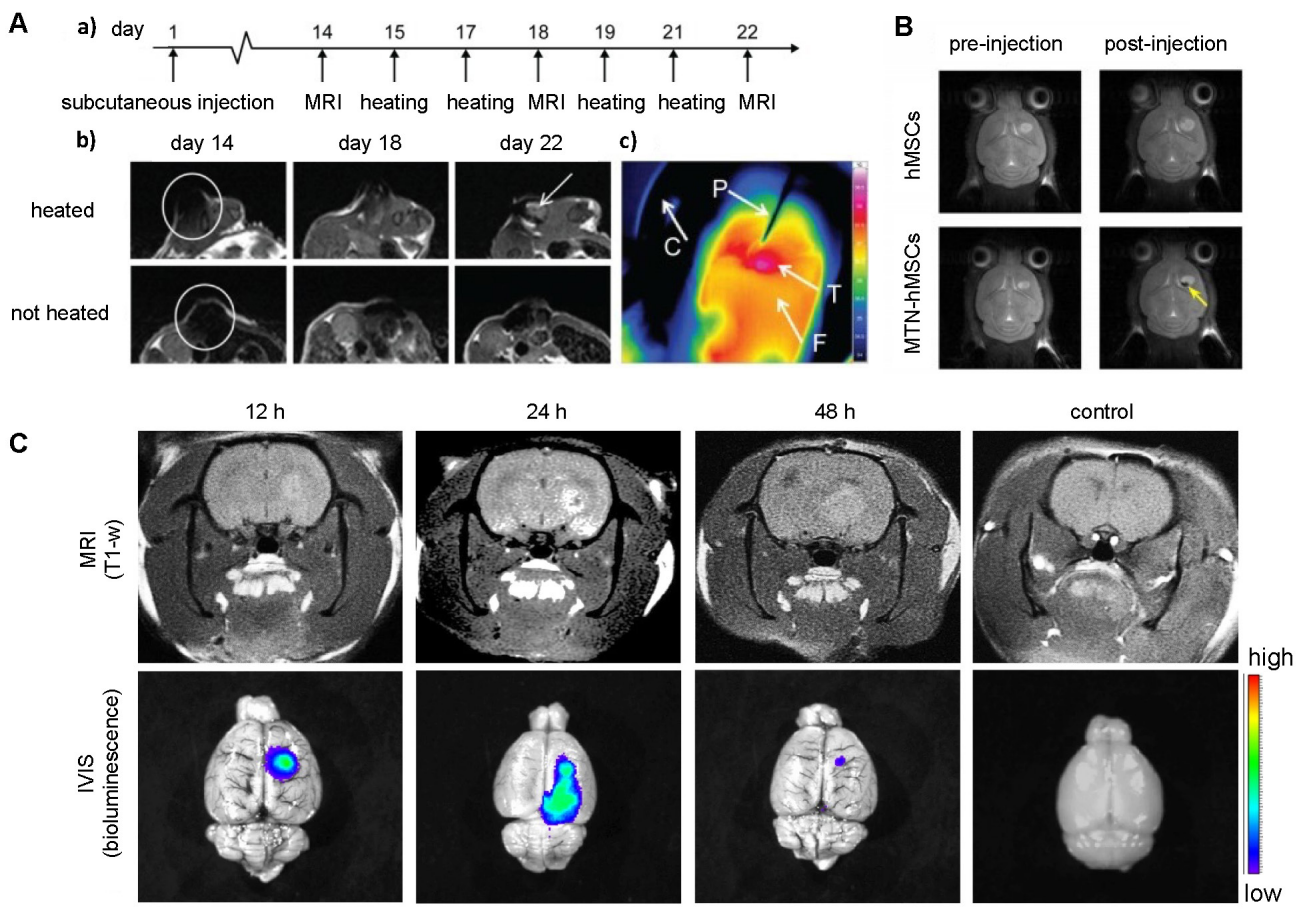
** (A)** T2-weighted MRI of co-injected ovarian cancer cells and SPIO-labeled MSCs 14 days post-injection (a, b) and heatmap of subsequent hyperthermia (c). Adapted from 88, courtesy of Dove Medical Press.** (B)** T2-weighted MRI of magnetic ternary hybrids complexed with TRAIL (MTN) 24 hours post intracerebral injection of MTN-labeled hMSCs. Yellow arrow indicates signal from labeled MSCs. Adapted from 89, courtesy of Ivyspring International Publisher. **(C)** T1-weighted MRI of Gd-containing NP-labeled MSCs 12, 24, and 48 hours post i.v. injection, with accompanying bioluminescence images. Adapted from 90, courtesy of Nature Springer.

To date, all cellular theranostics studies with T1- or T2-weighted MRI have mainly used SPIO- or Gd(III)-doped NPs as imaging agents. **Table 2** summarizes cellular nanotheranostics studies that used MRI as imaging modality. For most studies of MSC-mediated delivery of SPIO NPs, MRI was only applied to confirm successful tumor localization, without an attempt of therapeutic intervention. Kalber et al. visualized tumor localization of SPIO-labeled MSCs with MRI and then performed magnetic hyperthermia (**Figure 5A**). Surprisingly, they found no significant differences in tumor sizes after treatment, which may be due to either co-injection of SPIO-labeled MSCs with cancer cells, leading to inhomogeneous distribution of MSCs, or the low rise in surface temperature (4.3 C), which was insufficient to ablate surrounding cancer cells 88. Huang et al. showed that MSCs can be labeled with magnetic ternary nanohybrids (MTNs) containing SPIO and TNF-related apoptosis-inducing ligand (TRAIL) DNA for MRI and subsequent suicide gene therapy (**Figure 5B**) 89. Gd(III)-NP-labeled MSCs were used to target and image glioma with MRI, followed by Gd(III)-derived neutron capture therapy (**Figure 5C**) 90. All other studies with MSC-mediated delivery of Gd(III)-doped NPs have been limited to imaging. MRI was performed using Mn(II) and Gd(III) NP-labeled MSCs, with the cells locating in tumors after i.v. injection 72, 91, 92.

Multiple studies indicate that NP-labeled MSCs accumulate in tumors within 24 hours after i.v. injection 74, 92-94 and that they can be retained for up to 22 days with a visible MRI signal 88. However, the 22 day-study used a different approach: injection of an ovarian cancer cell line that was mixed with SPIO-labeled hMSCs to form subcutaneous tumors in mice 88. Other studies using i.v. injection observed that MSCs were retained for up to a week 95. Only two studies directly compared NP-labeled MSC accumulation and retention with naked NPs. Hao et al. injected SPIO NP-labeled MSCs and naked SPIOs i.v. in a breast cancer model, then performed MRI 1 hour post-injection - however, 1 hour is likely too short to achieve the full benefit from MSC-mediated delivery 96. Lai et al. performed MRI 48 hours post-i.v. injection of Gd(III)-containing NP-labeled MSCs and naked NPs in a glioma model, demonstrating that naked NPs could not reach the glioma while NP-labeled MSCs enabled MRI (**Figure 5C**) 90. No studies using i.v. or i.t. injection reported on the retention of NP-labeled MSCs for longer than one week, and hence, more studies on the long-term dynamics of NP-labeled MSCs are warranted. It would also be desirable to use MRI for comparison of the accumulation and retention of NP-labeled MSCs vs. naked NPs, as most studies thus far used only PBS or unlabeled MSCs as controls.

### 2.2. PAI

Cellular nanotheranostics imaged with PAI as a diagnostic modality have all used gold-based nanotheranostic agents (**Table 3**), including AuNPs in the form of nanostars 70, nanocages 97, nanorods 69, 98, and coated nanospheres 93. AuNPs are widely used as PAI contrast agents due to their strong and tunable optical absorption. When AuNPs interact with light, conduction electrons on the NP surface are driven by the incident electric field into collective oscillations, known as localized surface plasmon resonance (LSPR). After illumination, plasmons can decay nonradioactively or radioactively, resulting in absorption or light scattering, respectively 99. With PAI, the absorption cross-section of NPs can be measured after NIR light exposure, enabling deep tissue imaging and therapy guidance 27. In some studies, AuNPs were complexed with SPIO to allow dual-mode PAI and MRI of the same nanotheranostic agent 69, 93.

**Figure 6:**
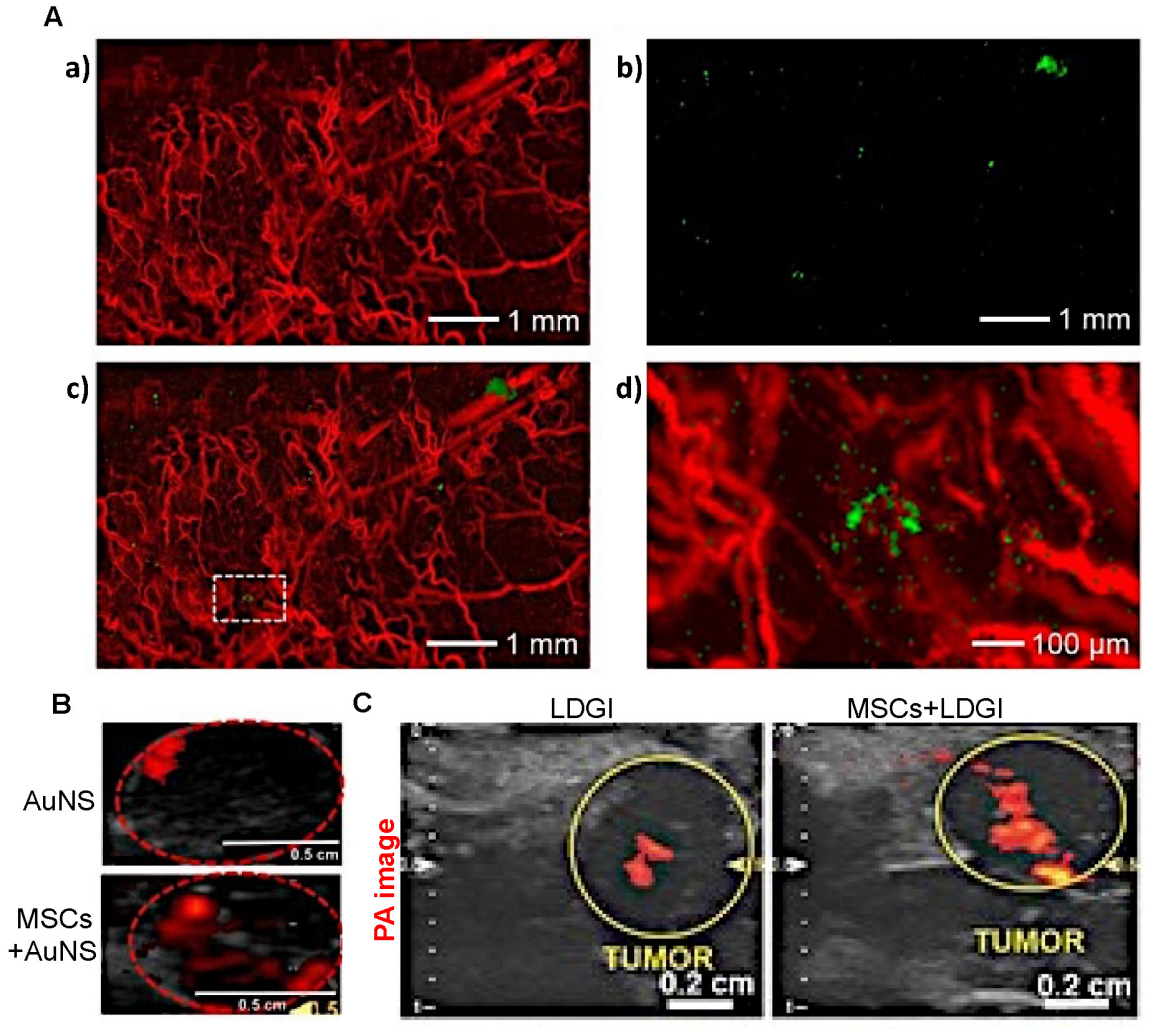
** (A)** AuNP-labeled MSCs (green) injected i.v. in a U87 brain tumor model are retained up to one-week post-injection. PA images show the presence of blood vessels (red) in the tumor acquired at a wavelength of 532 nm (a) and gold nanocage (AuNC)-labeled hMSCs injected i.v. that homed to the tumor region acquired at a wavelength of 638 nm (b). The images are superimposed in (c) with the inset shown at higher magnification in (d). Adapted from 97, courtesy of Ivyspring International Publisher.** (B)** PAI of gold nanostar (AuNS) and AuNS-labeled MSC distribution three days post i.t. injection, showing a 3.3-fold increase in signal area for MSC-mediated delivery. Adapted from 70, courtesy of Ivyspring International Publisher.** (C)** PAI of i.t.-injected nanoclusters composed of lipids, doxorubicin, gold nanorods, and iron oxide (LDGI) or LDGI-labeled MSCs, showing a 4.2-fold increase in signal area for MSC-mediated delivery. Adapted 69, courtesy of WILEY.

When imaged with PAI, i.v.-injected AuNP-labeled MSCs were observed to remain in tumors up to one week (**Figure 6A**) 97. Unfortunately, there are no comparison data available for retention of “naked” AuNPs for more than three days post-i.v. or i.t. injection and hence, it is not clear from these studies whether MSC-mediated delivery can improve long-term retention of PAI agents. However, with PAI, it was observed that MSC-mediated delivery markedly improved the distribution of AuNPs throughout tumors three days post i.t. injection, with a 3.3-fold (**Figure 6B**) 70 or 4.2-fold increase in area containing signal (**Figure 6C**) 69. Thus, MSC-mediated delivery can enable imaging of tumor areas that would otherwise not be visible with naked nanotheranostics.

### 2.3. Fluorescence imaging

Fluorescence imaging of cellular nanotheranostics has been primarily performed with NPs containing a photosensitizer including Ce6 100-102, dibenzocyclooctyne (DBCO)-fluorophores 71, and Cr(III) doped luminescent NPs 103. Alternatively, a few studies have used non-conventional fluorescent agents, such as Bi_2_Se_3_ NPs 104, Gd(III)-chelate NPs 72, and Mn(II) and Gd(III) co-doped CuInS_2_-ZnS nanocrystals 91 (**Table 4**).

**Figure 7:**
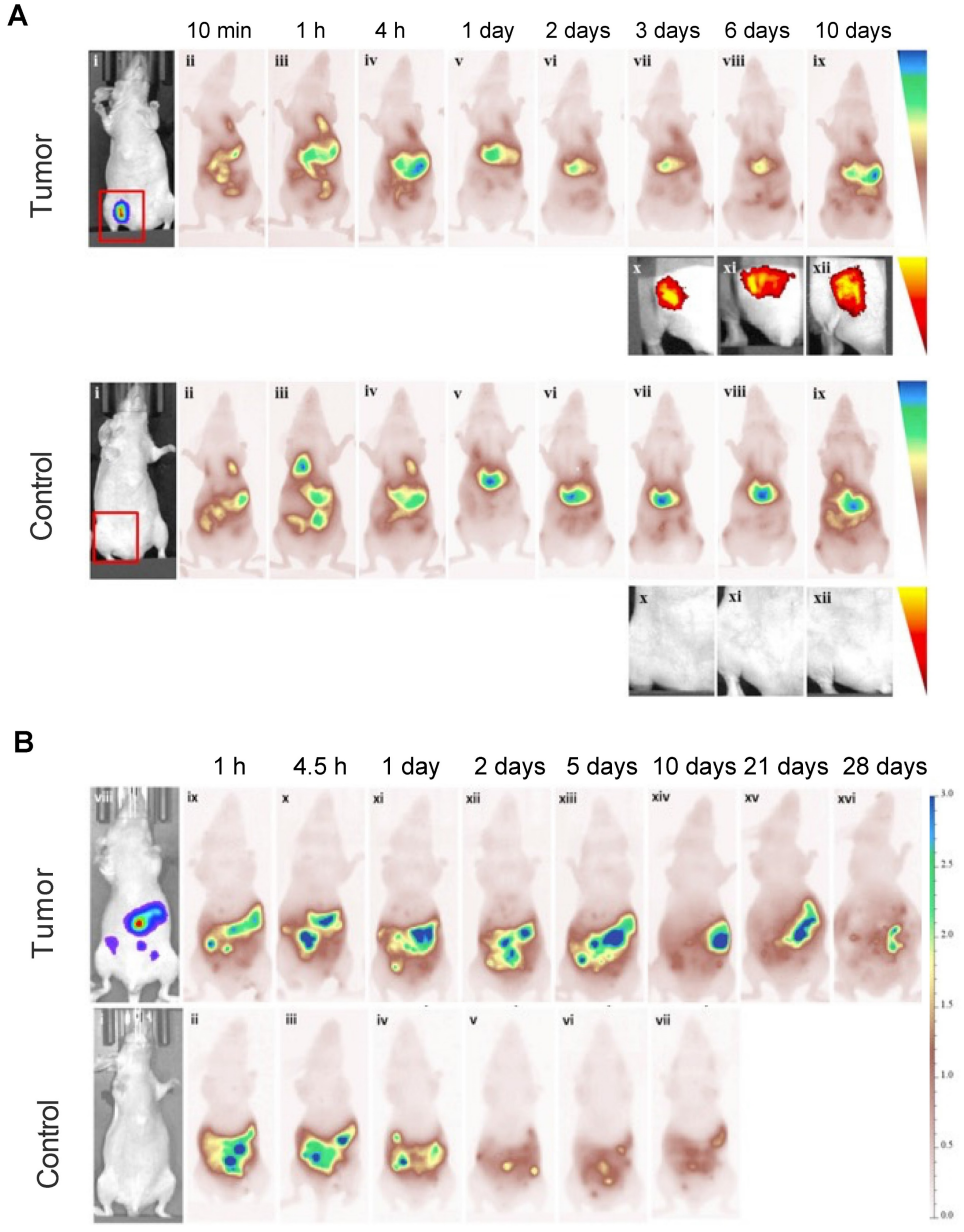
** (A)** Cy5.5-labeled MSCs injected i.v. in a lung carcinoma model, indicating tropism and retention of MSCs within tumors up to 10 days, along with non-specific cell distribution in the liver and spleen. Tumor-free mice were used as control. **(B)** Ovarian carcinoma model with i.p. injected Cy5.5-labeled MSCs, showing retention of Cy5.5-labeled MSCs within tumors up to 28 days. Tumor-free mice were used as control. Adapted with permission from 71, copyright 2016 Elsevier.

Studies performed with MSC-mediated delivery found that MSCs were retained within tumors for at least 10 or 28 days after i.v. and i.p. injection, respectively. At 10 days, the fluorescence intensity of Cy5.5-labeled MSCs was barely detectable in a lung carcinoma model, indicating that the majority of the Cy5.5 fluorophores were cleared by the liver and spleen (**Figure 7A**). A similar phenomenon was observed in an ovarian cancer model. On days 21 and 28 post i.p. injection, there was a small but detectable fluorescence signal, with most of the Cy5.5-labeled MSCs having cleared out the tumor (**Figure 7B**) 71.

Only one study has directly compared the distribution of Ce6-modified CdSe/ZnS QD (QD-Ce6)-labeled MSCs with QD-Ce6 alone for fluorescence imaging, reporting that there was a 7-fold higher fluorescence intensity when MSCs served as delivery vehicles for QD-Ce6 101. Overall, MSC-mediated delivery may significantly prolong the retention of nanotheranostics used for fluorescence imaging, enabling long-term monitoring of tumors during treatment. However, more studies are needed to compare the intratumoral distribution of nanotheranostic-labeled MSCs vs. naked nanotheranostics.

### 2.4. Other potential imaging modalities

Other imaging modalities may be used in the near future to monitor the biodistribution of cellular nanotheranostics, including MPI, magneto-motive ultrasound imaging (MMUS), and PET. MPI uses similar SPIO imaging agents as used in MRI, where they act as tracers instead of contrast agents 31. Unlike MRI, MPI can specifically quantify the amount of SPIO-labeled MSCs post administration 105, 106. MMUS can also use SPIO as an imaging agent 107, and hence has potential to detect SPIO-labeled MSCs as well. Finally, therapeutic NPs can be labeled with SPECT or PET tracers, but to the best of our knowledge no theranostic studies have been performed yet with NP-labeled MSCs.

## 3. Therapeutic modalities for cellular nanotheranostics

The primary therapeutic modalities used with cellular nanotheranostics include PTT 69, 70, 98 and PDT 100, 102, chemotherapy 71, 98, 108, and gene therapy 89, 103. PTT is performed with a near-infrared (NIR) laser beam directed at the tumor causing intratumoral NPs to heat up and ablate the tumor tissue 109. PDT is performed with an NIR laser as well, but it interacts with a PDT agent to produce single reactive oxygen species (ROS) and free radicals that lead to apoptosis, necrosis, and autophagy 110. Using appropriate photosensitizers, PDT has been used in the clinic for over forty years to treat a variety of tumors. Laser ablation without PTT agents has also been used clinically, although it has not reached large clinical trials. Neither PTT nor PDT with NPs has been clinically approved yet 109.

NP-based delivery of chemotherapeutics has been widely studied. NP vehicles currently undergoing clinical trials include liposomes, polymeric micelles, protein-drug NPs, and dendrimers 111. The efficacy of NPs as chemotherapeutic carriers to tumors depends on the EPR effect and rate of controlled drug release 112. Therapeutic genes can be delivered with viral vectors or NPs. NPs have advantages as delivery agents due to their comparatively lower toxicity and higher carrying capacity 25. While gene therapy has not been clinically approved as a form of cancer therapy, many studies have indicated that NPs are promising delivery agents in experimental animal models 113, 114. In the following sections, we provide an overview of current approaches towards tumor therapy employing MSC-mediated delivery of therapeutic agents.

### 3.1. PTT and PDT

Cellular nanotheranostics with PTT as a therapeutic modality have primarily used Au-based NPs as photothermal agents. To enhance PTT heating, the shape and surrounding materials of AuNPs can be optimized, as demonstrated for gold nanostars (AuNS) 70, gold nanorod-embedded hollow periodic mesoporous organsilica nanospheres (AuNR-HPMOs) 98, and gold nanorod (AuNR) and iron oxide (LDGI) nanostructures 69. With AuNPs being an excellent imaging agent for PAI, it is not surprising that all cellular nanotheranostic PTT studies have used PAI as an imaging modality (**Table 5**).

**Figure 8:**
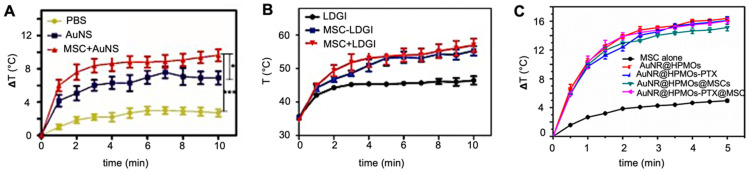
** (A)** Temperature changes during irradiation of prostate tumors post-i.t. injection of naked gold nanostars (AuNS) and AuNS-labeled MSCs, showing a differential temperature increase of approximately 2°C for MSC-mediated delivery. Adapted from 70, courtesy of Ivyspring International Publisher.** (B)** Temperature changes during irradiation of breast tumors post-i.v. injection of nanoclusters composed of lipids, doxorubicin, gold nanorods, and iron oxide (LDGI), LDGI-labeled MSCs, and LDGI-labeled MSCs, with a differential temperature increase of approximately 13°C for MSC-mediated delivery. Adapted from 69, courtesy of WILEY.** (C)** Temperature changes during irradiation of breast tumors post-i.t. injection of gold nanorod-embedded hollow periodic mesoporous organosilica NPs loaded with paclitaxel (AuNR@HPMOs-PTX) and AuNR@HPMO-PTX-labeled MSCs. Adapted with permission from 98, copyright 2016 ACS Publications.

Huang et al. found a comparative temperature increase of approximately 2°C three days post i.t. injection for MSC-mediated delivery of AuNS (**Figure 8A**) 70, while Xu et al. found an increase of no less than 13°C three days post i.v. injection of AuNR-labeled MSCs (**Figure 8B**) 69. Xu et al. also found that tumors treated with AuNR-labeled MSCs and PTT had ultimately no increase in volume, while tumors injected with naked AuNRs had a six-fold volume increase 69. Despite the disparity in temperature difference, both Huang's and Xu's team used similar PTT conditions, including time post-injection, number of hMSCs, PTT agent injected, and laser power. The primary difference here was the injection route, i.e., i.v. vs. i.t. When naked AuNRs are injected i.v., fewer particles are expected to reach the tumor compared to AuNRs internalized and delivered by tumor-tropic hMSCs. Wu et al. exposed tumors to a PTT laser 24 hours post-i.t. injection and observed similar temperature increases for AuNRs delivered by MSCs vs. alone as naked AuNRs **(Figure 8C)**. It is possible that at the 24-hour time point, neither nanotheranostic was cleared out yet from the tumor, making them both efficient PTT agents 98.

Cellular nanotheranostics using PDT as therapeutic modality have all used Ce6 as the photosensitizing molecule with fluorescence imaging as modality. Similar to PTT, the observed tumor volume decrease was most pronounced for MSC-mediated delivery when PDT was performed after i.v. injection or delayed after i.t. injection. PDT performed 24- and 48 hours post-i.t. injection resulted in a 4-fold reduced tumor size for MSC-mediated delivery compared to naked NPs 101. In contrast, only a 1.25-fold reduced tumor size for MSC-mediated delivery vs. naked NPs was observed when PDT was performed immediately after i.t. injection 102. Overall, the longer the delay between i.t. injection and therapy, the more efficient MSC-delivered therapy becomes compared to naked NPs. This suggests that MSC-mediated delivery of nanotheranostics may require only a single injection, which can be used for multiple rounds of therapy spread out over time.

### 3.2. Chemotherapy

**Figure 9:**
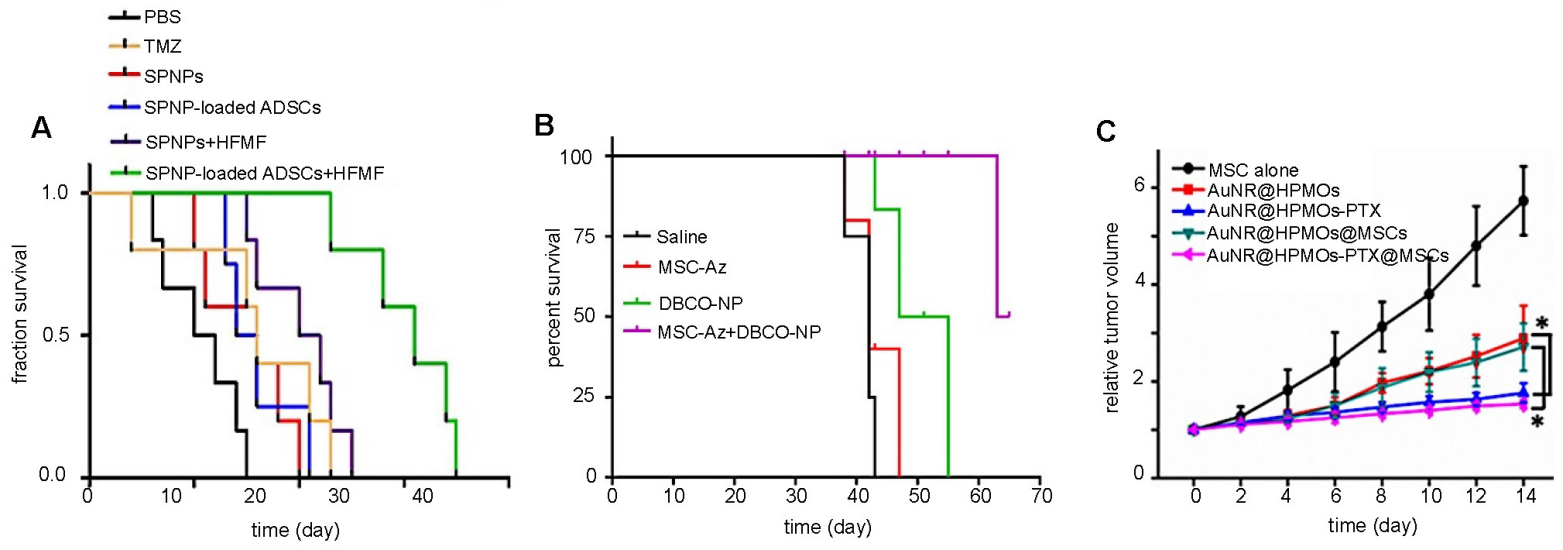
** (A)** Survival rates of brain tumor-bearing mice injected i.v. with naked SPIO/paclitaxel (PTX)-loaded polymeric NPs (SPNPs) and SPNP-loaded MSCs, with and without hyperthermia. Adapted with permission from 115, copyright 2017 Elsevier.** (B)** Survival rates of ovarian carcinoma-bearing mice injected i.p. with paclitaxel-loaded, DBCO surface functionalized NPs (DBCO-NPs) and DBCO-NP-labeled glycoengineered MSCs. Adapted with permission from 71, copyright 2016 Elsevier.** (C)** Relative breast tumor volume of mice injected i.t. with gold nanorod-embedded hollow periodic mesoporous organosilica NPs loaded with paclitaxel (AuNR@HPMOs-PTX) and AuNR@HPMO-PTX-labeled MSCs. Adapted with permission from 98, copyright 2016 ACS Publications.

An overview of cellular nanotheranostic agents used in tandem with chemotherapy as therapeutic modality is listed in **Table 6**. All nanotheranostics incorporated the chemotherapeutic drug paclitaxel (PTX). Imaging agents include SPIO 115, DBCO-labeled fluorophores 71, 108, and AuNR@HPMOs 98, using MRI, fluorescence imaging, and PAI, as imaging modalities, respectively. Overall, MSC-mediated delivery of PTX-loaded nanotheranostics increased mice survival rates compared to PTX alone, although it should be noted that in some studies MSC-PTX was combined with hyperthermia 115 and PTT, respectively 98, making this an improper comparison. MSC-mediated delivery of nanochemotheranostic agents remains promising (**Figure 9**) but further studies are needed on its overall efficacy.

### 3.3. Gene therapy

**Figure 10:**
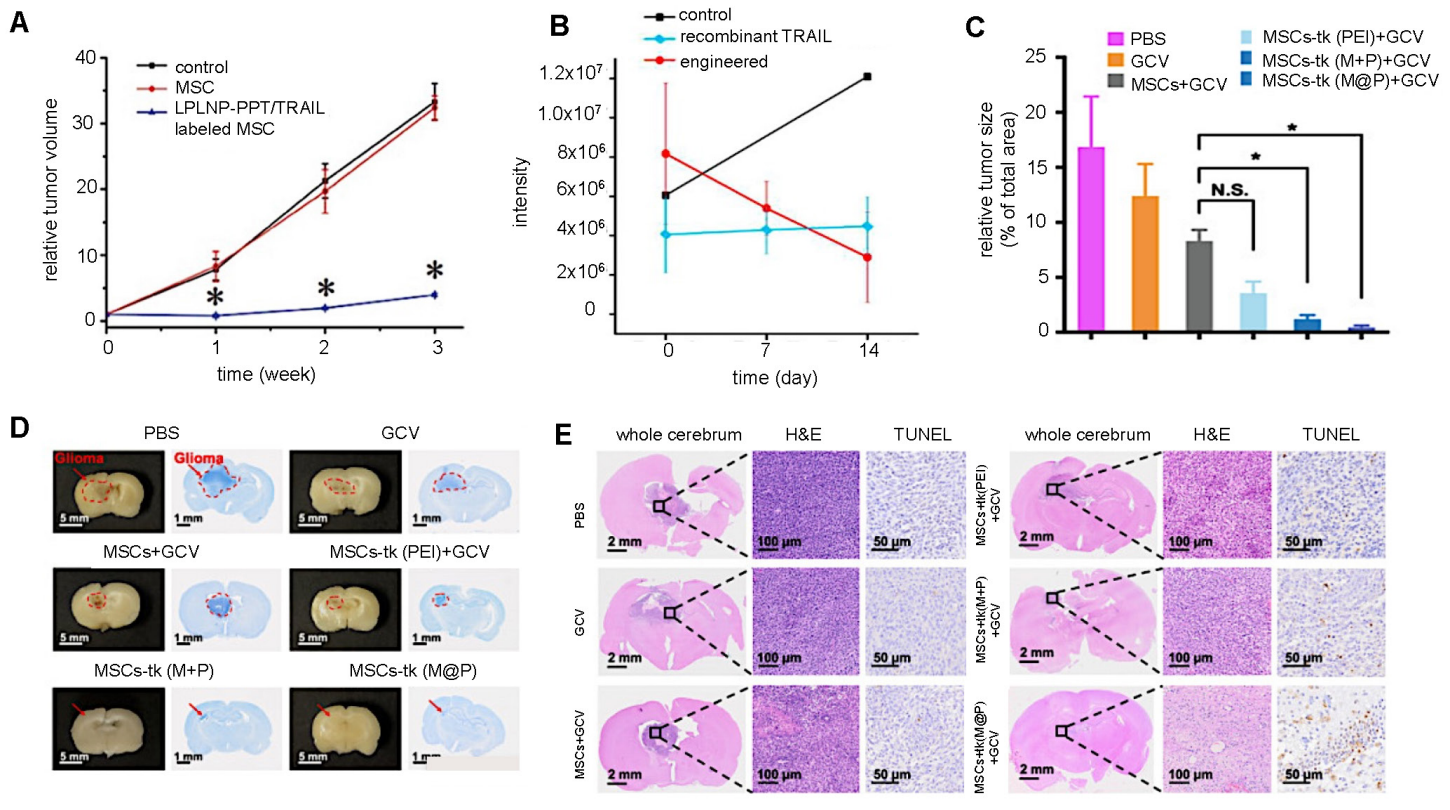
** (A)** Relative brain tumor volumes of mice injected i.t. with MSCs and MSCs labeled with long persistent luminescence NPs complexed with TRAIL (LPLNP-PPT/TRAIL). Adapted with permission from 103, copyright 2017 WILEY. **(B)** Luminescence intensity quantification of luciferase-expressing ovarian tumor injected i.p. with recombinant TRAIL protein and magnetic NP-PEI/TRAIL plasmid complexes. Adapted with permission from 117, copyright 2016 Elsevier. **(C, D, and E)** Relative tumor size and brain sections stained with Nissl, H&E, and TUNEL for gliomas injected i.v. with herpes simplex virus thymidine kinase (HSV-tk)-transduced MSCs. Ganciclovir (GCV) was administered to kill tumor cells after phosphorylation by HSV-tk. Adapted from 116, courtesy of Ivyspring International Publisher.

Most cellular nanotheranostic agents using gene therapy are composed of magnetic NPs using MRI as the imaging modality (**Table 7**). The structural composition of these NPs included magnetosome-like ferrimagnetic iron oxide nanochains (MFIONs) 116, ZnFe_2_O_4_ magnetic core and mesoporous silica shell (MCNPs) 117, and a magnetic ternary nanohybrid (MTN) system comprised of cationic materials, nucleic acids, and hyaluronic acid-decorated SPIO 89. Only one study used fluorescence imaging as the imaging modality with dual-functional persistent luminescent nanocomposites (LPLNP-PTT/TRAIL) 103. Here, hMSCs were transfected with TRAIL DNA (**Figure 10A**). Once localized in tumors, TRAIL-hMSCs secrete TRAIL, which binds to the death receptor 4 (DR4) or DR5 of tumor cells, causing apoptosis 118. Only Yin et al. compared MSC-mediated delivery of MCNP-TRAIL plasmid complexes with intraperitoneal (i.p.) injection of TRAIL alone (**Figure 10B**) 117. Ovarian tumors treated with MSCs engineered by MCNP-TRAIL plasmid complexes had a volume decrease of over 50% after two weeks, while tumors that received a single dose of TRAIL had no volume decrease. This may be explained by the short half-life of TRAIL, which typically requires high daily doses (1-10 mg/kg) to be effective 119. No comparison was done for treatment efficacy of naked MCNP-TRAIL plasmid complexes. All studies used TRAIL for gene therapy, except for Li et al. who elected to use the herpes simplex virus thymidine kinase (HSV-tk)/ganciclovir (GCV) suicide gene for glioma treatment (**Figure 10C-E**) 116.

Although combining cellular nanotheranostics with gene therapy has so far only been applied for TRAIL and HSV-tk/GCV genes, previous studies have indicated that MSCs can be engineered to express a wide range of genes for therapeutic purposes. MSCs expressing immunomodulatory cytokines such as IFN-β 120 and IL-12 121, 122 inhibited tumor growth in melanoma, renal cell carcinoma, and cervical cancer murine models. Genes for the anti-angiogenic protein thrombospondin (TSP-1) 123 and the suicide gene cytosine deaminase-uracil phosphoribosyl transferase (CD-URBT) 124-126 prevented cancer progression in glioma, melanoma, prostate, and colon cancer murine models. In future studies, the use of genetically engineered cellular nanotheranostics may be used for further improvement of NP delivery.

### 3.4. Radiation therapy

**Figure 11:**
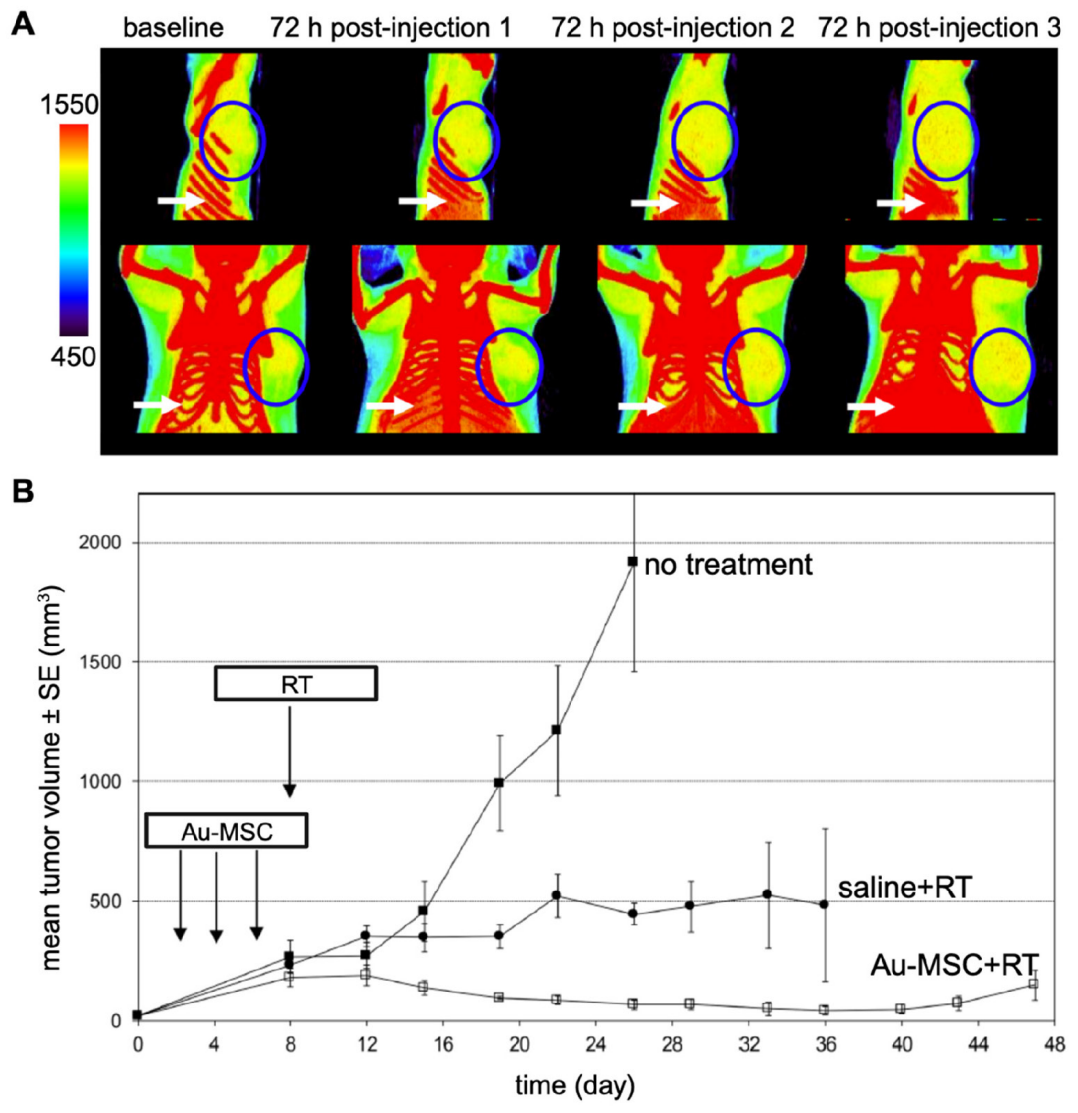
** (A)** CT images of mice bearing MDA-MB-231 tumors taken 72 hours after each i.v. injection of AuNP-labeled MSCs. Tumors are indicated by blue circles, with red pixels within the circles representing the accumulation of high-density particles at the tumor site. CT images show the accumulation of labeled MSCs in the liver and spleen (white arrows). **(B)** Treatment outcome in NOD/SCID mice bearing subcutaneous MDA-MB-231 tumors. Adapted from 128, courtesy of MDPI.

Nanoparticle-mediated radiotherapy (RT) has considerable potential for revolutionizing cancer treatment 127, particularly when combined with MSCs for targeted NP tumor delivery 128. The strategic integration of MSCs enhances precision in cancer radiotherapy by providing a specialized vehicle for the transport of radiosensitizing NPs to the tumor. When guided by either MRI or CT, the entire procedure can be personalized. Lai et al. 90 used MSCs to deliver GD(III)-based NPs across the blood-brain-barrier to gliomas after i.v. injection. MRI and gadolinium neutron capture therapy was then performed, reducing tumor volume 4-fold and increasing median survival 2.5-fold compared to gadolinium NPs alone. Xiao et al. 129 investigated the potential of tumor-tropic adipose-derived MSCs for targeted RT of NSCLC using bismuth selenide (Bi_2_Se_3_) NPs. Key findings included the ability of MSCs to selectively deliver Bi_2_Se_3_ NPs to NSCLC tumors following i.v. injection, the enhancement of radiation-induced cell death, the significant reduction in tumor growth and prolongation of animal survival after RT, the increased radiation-induced apoptosis and reduced tumor angiogenesis associated with the therapeutic efficacy, and the favorable safety profile of MSCs-mediated Bi_2_Se_3_ NP delivery. In another study, Pullambhatla et al. 128 demonstrated that RT is more effective when paired with AuNP-labeled MSCs. Since AuNPs can enhance CT image contrast, mice bearing MDA-MB-231 breast tumors were imaged with CT before injection of labeled MSCs and at 3 different time points after (**Figure 11**). Following imaging at baseline, mice were injected i.v. with labeled MSCs on days 0, 3, and 6 for a total of 3 injections administered 72 hours apart. Labeled MSCs demonstrated accumulation at the tumor site at 72 hours post injection, which increased progressively after successive injections of labeled MSCs (**Figure 11A**). Two days after injection of labeled MSCs had been completed (day 8), mice were irradiated under CT guidance. Without treatment the mean tumor volume was 2,000 mm^3^ at day 24 following tumor inoculation, while mice treated with labeled MSCs in combination with radiotherapy exhibited the greatest delay in tumor growth, with a mean tumor volume of only 15 mm^3^ at day 45 post tumor inoculation (**Figure 11B**).

Although more studies are required, these initial reports suggest that the utilization of imaging-guided nanoparticle-mediated RT combined with MSCs as delivery vehicle presents a promising new treatment strategy.

### 3.5. Other potential therapeutic modalities

Cellular nanotheranostics may be further engineered based on other modes of cancer therapy. Stem cells engineered for cancer immunotherapy are a promising avenue for cellular nanotheranostics 130. For instance, Zhang et al. incorporated methylene blue as a photosensitizer in MSCs transfected with a plasmid encoding IL-12 and were able to demonstrate an enhanced immune response after PDT 131. Furthermore, it has been shown that after RT-induced tumor ablation, there is a release of tumor-associated antigens which modulates the efficacy of immunotherapy, including cytotoxic T-lymphocyte associated protein 4 (CTLA-4) checkpoint blockade 132 and anti-programmed cell death ligand 1 (PD-L1)-based immunotherapy 133. Hence, NPs could be potentially targeted to multiple tumor metastases if delivered using MSCs, allowing extensive RT and enhancing subsequent immunotherapy.

Hematopoietic stem cell transplantation (HSCT) has been widely used for haematological malignancies and solid tumors, allowing patients to effectively regenerate hematopoietic cells after high-dose chemotherapy 134, 135 or RT 136. Cluster of differentiation 19 (CD19) CAR-T cell therapy has been combined with HSCT, where CD19 CAR-T cell therapy eradicates leukemia cells and B cells, and HSCs promote the amplification and survival of CD19 CAR-T cells 137, 138. HSCs could be labeled with nanotheranostic agents to simultaneously visualize their localization at the transplantation site and other tissues.

Finally, hormone therapy, which acts by blocking the receptors of endocrine cancers and prevent further cell proliferation, has been combined with NPs for enhanced imaging and therapy of cancer 139, 140. MSCs could be possible genetically engineered to increase hormone production while also labeled with NPs.

## 4. Some critical notes and challenges to move forward

The future clinical use of cellular nanotheranostics faces many challenges. As for optimal timing for performing PTT, hyperthermia, and/or RT for example, the (long-term) duration of the retention of MSCs within tumors has been evaluated only a few studies. Near all studies followed up for only a few hours post-injection, with a maximum of one week. However, some studies reported that MSCs, once homed to tumors, can remain there for 3-4 weeks. Most studies only compared MSCs alone with MSC-mediated delivery of nanotheranostics. Since MSCs (without labeling) may be effective cellular therapeutics by themselves, using them as the only control is not a proper way to validate their promise as delivery vehicles of nanotheranostics 141-143.

Each cancer, depending on where it originated, has a different molecular signature, vascularization, and aggressiveness, which may influence MSC homing 144. Only one study has made a direct comparison of the efficacy of cellular nanotheranostics towards different tumor types, reporting that Cy5.5-labeled MSCs were retained in lung cancer models for up to 10 days and in ovarian cancer models for up to 28 days 71. Also, it remains unclear what the effect of tumor size is on stem cell tropism, although past studies demonstrated that stem cells can successfully 'trail' small glioma metastases 145.

After i.v. injection, MSCs can home into the tumor but also in other tissues that are wounded or renewing quickly, a hallmark of natural MSC repair 146, 147. To maximize their therapeutic potential, methods for enhancing MSC tumor-tropism must be further developed so that MSCs can more specifically target tumors. Such approaches include genetic engineering to overexpress the chemokine receptors CXCR1-4 148, 149 IL-8 150, and interferon-β 120. In the last study, a therapeutic effect was not observed for systemic delivery of IFN-β or by IFN-β produced by MSCs injected subcutaneously at a site distant from the tumors. Another example how cells can be engineered to improve their initial docking to target tissue is to transfect them with the very late antigen-4 (VLA-4), which specifically binds to the vascular cellular adhesion molecule 1 (VCAM-1) expressed on inflamed endothelium 151. This facilitates their extravasation and passage into the brain parenchyma 152. Of note, it has been demonstrated that pre-treating tumors with RT can improve MSC recruitment 153-155.

It remains controversial whether MSCs promote tumor progression and metastasis 156, 157 or enhance pathways that suppress both proliferation and apoptosis 158, 159. To safely administer cellular nanotheranostics for cancer, it is essential to avoid MSCs becoming pro-carcinogenic. For example, for the same breast cancer cell line MDA-MB-231, injected MSCs have been reported to either promote tumor metastasis 62, 160 or inhibit primary tumor progression to metastasis 161. Several studies which claimed tumorigenic effects of MSCs *in vitro* and *in vivo* have been retracted due to cross-contamination with cancer cell lines, making a conclusive evaluation difficult 162. MSCs have been reported to inhibit cancer progression in glioblastoma 158, leukemia 163, and hepatoma 159 murine models. Thus, further investigations are needed to determine the clinical efficacy and safety of MSC-mediated nanotheranostic delivery for cancer therapy.

Finally, MSC-derived extracellular vesicles (EVs) may be a potential alternative to using parental MSCs as nanotheranostic delivery agents by incorporating imaging agents 164. It is not yet clear if such an approach would eliminate the risks associated with intact MSC delivery, as related to MSCs promoting tumor progression 66. However, a key drawback of using EVs instead include the difficulty of loading them and the lack of standardized production and purification methods 165.

## Conclusions

MSC-based cellular nanotheranostics with follow-up imaging of delivery and retention may become a promising new method for treating cancer. Recent progress has demonstrated this to work well in pre-clinical cancer models for a diverse set of imaging and therapeutic modalities, including those that are clinically practiced such as MRI and chemotherapy. MRI has confirmed successful targeting of tumors when cellular nanotheranostics are employed, an objective that is difficult to achieve with traditional “naked” nanotheranostics. PAI and fluorescence imaging have also demonstrated that cellular nanotheranostics are able to deliver and retain nanotheranostic agents within tumors, enabling long-term comprehensive imaging. The enhanced delivery, distribution, and retention accomplished with cellular nanotheranostics will lead to an improved therapeutic outcome. PTT and PDT performed after MSC-mediated delivery of nanotheranostics indicate reduced tumor volumes and increased survival rates compared to administration of naked nanotheranostics. Chemotherapy and gene therapy can be used to increase survival rates when combined with cellular nanotheranostics.

Furthermore, MSC-based cellular nanotheranostics may target hypoxic tumors that are otherwise resistant to chemotherapy, RT, and immunotherapy. However, phase I clinical trials have indicated that a key challenge for MSC-mediated delivery may be an insufficient accumulation of MSCs within tumors post-i.v. administration. When delivered via an intraperitoneal catheter, MSCs successfully targeted ovarian tumors 166, but when administered systematically, which is clinically preferred, MSCs did not reach primary prostate tumors 167. To achieve the full potential of MSC-based cellular nanotheranostics, it is imperative for future studies to elucidate the relative significance of cytokines, chemokines, and growth factors implicated in MSC tumor-tropism in inducing signaling pathways associated with cell migration. Additionally, a comprehensive understanding is needed of the temporal coordination of the signaling pathways involved in MSC tumor-tropism. Once the mechanism of MSC tumor-tropism is much better understood, MSCs may be further genetically modified to enhance tumor targeting, and thus their efficacy as delivery agents of nanotheranostics.

## Figures and Tables

**Table 1:** Postulated signaling pathways and molecular mechanisms of MSC tumor-tropism.

Signaling molecule	Signaling pathways in MSCs	Mechanism for MSC tumor-tropism
TNF-α	TNF-α induces VCAM-1 and VLA-1 expression on MSCs through the PI3K- and NF-kB signaling pathways. MSC VCAM-1 expression is much higher than VLA-1 40. VCAM-1 and VLA-1 expressing MSCs adhere to endothelial cells, although the exact biological mechanism of this remains unknown 41. TNF-α also causes MSCs to secrete MMP-1, which cleaves extracellular IGF-2/IFGBP-2 complexes 42. Free IGF-2 is released from the complex and binds to the MSC receptors IGF-1R or IR-A, inducing activation of PI3K-Akt/PKB and MAPK. It is unclear how these pathways interact to promote MSC migration 43.	Enables adhesion to tumor vessel endothelial cells and cleavage of extracellular IGF-2/IGFBP-2 complexes, activating IGF-2 signaling pathways that promote MSC migration.
IL-6	IL-6 binds to IL-6 receptors and GP130 on MSCs, inducing CXCL7 expression and STAT3 activation 44. MSC-derived CXCL7 interacts with cancer cells through the CXCR2 receptor, inducing tumors to further synthesize IL-6 and IL-8. STAT3 activation, resulting from tumor-secreted IL-6 that binds to IL-6R on MSCs, induces the NF-kB-IL-6-STAT3 cascade, which leads to modest activation of ERK and cytoskeletal longitudinal organization of MSCs, enhancing the cell migratory phenotype 45.	Induces an inflammatory cytokine feedback loop and MSC migration phenotype (ERK activation and cytoskeletal organization).
IL-8	IL-8 binds to CXCR1 46 and CXCR2 47 on MSCs. The intracellular signaling pathway induced by CXCR1 binding is unknown, although it has been found to play a significant role in MSC tumor-tropism. CXCR2 binding leads to Akt and ERK phosphorylation, potentially activating the respective intracellular pathways and subsequent MSC migration 48.	Activates intracellular pathways (Akt and ERK) associated with MSC migration.
SDF-1	SDF-1 binds to CXCR4 on MSCs 49. In response, MSCs secrete more SDF-1, which acts in an autocrine manner. SDF-1 binding to CXCR4 activates downstream JAK2/STAT3 50 and ERK/MAPK 51 signaling in MSCs. FAK and paxillin signaling is then activated, which induces cytoskeletal reorganization.	Induces SDF-1 production and MSC migration phenotype (JAK2/STAT3 and ERK/MAPK pathways activated, cytoskeletal organization).
CXCL1	The CXCL1 ligand binds to CXCR2 receptors on MSCs 52, 53. The exact signaling pathways induced in tumor-tropic MSCs after CXCR2 binding are unknown, but previous studies have indicated that CXCR2 binding can activate the PI3K-Akt/PKB, ERK, MAPK, JAK2, and STAT3 pathways in other cells 54.	Unknown role for inducing MSC migration.
MCP-1	MCP-1 binds to the CCR2 receptor on MSCs, inducing unknown intracellular signaling pathways to promote migration 55. The stromal population of cells adjacent to tumors, consisting of fibroblasts, are the main source of MCP-1 secretion.	Unknown role for inducing MSC migration.
TGF-β	TGF-β binds to TGF-β receptors on MSCs, upregulating CXCR4 and inducing lamellipodia protrusions 56. Other studies indicate that SDF-1 binds to CXCR4, so it is plausible that through CXCR4 upregulation, TGF-β indirectly activates JAK2/STAT3 and ERK/MAPK signaling in MSCs, which then induces cytoskeletal reorganization 57.	Induces CXCR4 upregulation and MSC migration phenotype (lamellipodia protrusions).
PDGF	PDGF binds to PDGF receptors on MSCs 58, activating the PI3K pathway and subsequent MSC migration 59.	Activates an intracellular pathway (PI3-kinase) associated with MSC migration.
HIF-1α PGF CXCL16 CSF-1	Cancer cells expressing HIF-1α secrete PGF, CXCL16 60, and CSF-1 61, as well as the receptors CXCR3 and CCR5. PGF and CXCL16 bind to respective VEGFR1 and CXCR6 receptors on MSCs, which cause MSCs to secrete CXCL10. CSF-1 binds to the CSF-1 receptor on MSCs, inducing CCL5 secretion. CXCL10 and CCL5 bind to CXCR3 and CCR5 receptors on cancer cells, respectively. MSCs stimulate further HIF-1α expression in cancer cells through a CXCR3-independent mechanism 62. The intracellular signaling pathways induced by CXCR3, CXCR6, VEGFR1, and CSF-1R binding of MSCs, and how they promote tumor-tropism, remain unclear.	Induces chemokine and cytokine interactions between cancer cells and MSCs, as well as increases HIF-1α production in cancer cells. Unknown role for inducing MSC migration.

Abbreviations: Akt=Ak strain transforming; CCR=CC motif chemokine receptor; CSF=colony stimulating factor; CXCL=chemokine (C-X-C motif) ligand; CXCR= chemokine (C-X-C motif) receptor; FAK=focal adhesion kinase; ERK=extracellular signal-regulated kinase; HIF=hypoxia inducible factor; ICAM=intercellular adhesion molecule; IGF=insulin-like growth factor; IFGBP=insulin-like growth factor binding protein; IL=interleukin; JAK=janus kinase; MAPK=mitogen-activated protein kinase; MCP=monocyte chemoattractant protein; MMP=matrix metallopeptidase; NF=nuclear factor; PDGF=platelet-derived growth factor; PGF=placenta growth factor; PI=phosphoinositide 3-kinase; PKB SDF=stromal cell-derived factor; STAT=signal transducer and transcription activator; TGF=transforming growth factor; TNF=tumor necrosis factor; VCAM=vascular cell adhesion molecule; VEGF=vascular endothelial growth factor; VLA=very late antigen.

**Table 2:** Nanotheranostic MSC studies that have used MRI as imaging modality.

MSC origin	Cancer type (cell line)	Animal model	Imaging agent	Injection route and n of labeled cells	Imaging paradigm				Retention time of MSC-NPs	Retention time of naked NPs	Reference
					Field strength	Contrast	Time post-injection	Control			
Bone marrow	Glioblastoma (C6)	Wistar rats	SPIO	I.v., 1.5E5	7.1 T	T2-w	24 h	PBS	>24 h	N/A	94
N/A	Ovarian cancer (OVCAR-3)	Immunosuppressed BALB/c nu/nu mice	SPIO	I.v., 5E5	9.4 T	T2-w	14,18,21 d	PBS	22 d	N/A	88
Bone marrow	Breast cancer (4T1)	ICR mice	SPIO	I.v., N/A	N/A	T1-w	1 h	Naked SPIO	>1 h	>1 hour	96
Bone marrow	Glioblastoma (U87MG)	Athymic nude mice	Zinc-doped SPIO	I.v., 1E6	7 T	T1-w, T2-w	48 h	Unlabeled MSCs	>48 h	N/A	74
Bone marrow	Glioblastoma (U87MG)	BALB/c nude mice	PEG-SPIO	I.v., N/A	7 T	T2-w	7 d	Tumor-free	>7 d	N/A	95
N/A	Glioblastoma (U87MG)	Athymic nude mice	SPIO@Au NPs	I.v., 1E6	7 T	T2-w	72 h	Unlabeled MSCs	>3 d	N/A	93
Bone marrow	Glioblastoma (U87MG)	BALB/c mice	Silica-Gd	I.v., 1E6	7.05 T	T1-w	24 h	Naked NP	>24 h	N/A	92
Bone marrow	Colon cancer (CT26)	BALB/c nude mice	Gd(III)-chelate NPs	I.v., 1E5	4.7 T	T1-w	2 h	Unlabeled MSCs	>2 h	N/A	72
Umbilical cord	Glioma (GBM8401)	F344/NNarl rat; C57BL/6JNarl rat	Gd-SPIO	I.v., 2E6	3 T	T2-w	48 h	Naked NP	>48 h	N/A	90
Umbilical cord	Melanoma (B16F10)	C57BL/6 mice	Mn(II) and Gd(III) co-doped CuInS2-ZnS nanocrystals	I.v., 1E6	1.5 T	T1-w, T2-w	6 h	PBS	>6 h	N/A	91

Abbreviations: I.t.=Intratumoral; I.v.=Intravenous; MSC=Mesenchymal stem cell; N/A=Not available; NP=Nanoparticle; PEG=Polyethylene glycol; SPIO=Superparamagnetic iron oxide

**Table 3:** Nanotheranostic MSC studies that have used PAI as imaging modality.

MSC origin	Cancer type (cell line)	Animal model	Imaging agent	Injection route and n of labeled cells	Imaging paradigm			Retention time of MSC-NPs	Retention time of naked NPs	Distribution area of MSC-NPs	Distribution area of naked NPs	Reference
					Wavelength	Time (post- injection)	Control					
Umbilical cord	Prostate (PC-3)	Nude mice	AuNS (anisotropic)	I.t., 1E5	780 nm	3 d	Naked AuNS	>3 d	>3 d	0.073 cm^2^	0.022 cm^2^	70
Bone marrow	Glioblastoma (U87-MG)	Athymic nude mice	AuNC (anisotropic)	I.v., 1E5	638 nm	7 d	N/A	>7 d	N/A	200 um^2^	N/A	97
Bone marrow	Glioblastoma (U87-MG)	Athymic nude mice	SPIO@Au	I.v., 1E6	810 nm	3 d	Unlabeled MSCs	>3 d	N/A	N/A	N/A	93
Umbilical cord	Negative breast cancer (MDA-MB-231)	BALB/c athymic nude mice	LDGI	I.t., 1E5	820 nm	3 d	Naked LDGI	>3 d	>3 d	0.0945 cm^2^	0.0225 cm^2^	69
N/A	Breast cancer (MCF-7)	BALB/c nude mice	AuNR@HPMOs-PTX	I.t., 1E6	700 nm	24 h	AuNR@HPMOs-PTX	>24 h	>24 h	N/A	N/A	98

Abbreviations: AuNC=Gold nanocages; AuNR@HPMOs-PTX=Gold nanorod hollow periodic mesoporous organosilica nanospheres with paclitaxel; AuNS=Gold nanostar; I.t.=Intratumoral; I.v.=Intravenous; LDGI=Lipid-Doxorubicin-Gold-Iron oxide nanocluster; MSC=Mesenchymal stem cell; N/A=Not available; NP=Nanoparticle; SPIO=Superparamagnetic iron oxide

**Table 4:** Nanotheranostic studies that have used fluorescence imaging as imaging modality.

MSC origin	Cancer type (cell line)	Animal model	Imaging agent	Injection route and n of labeled cells	Imaging paradigm			Retention of MSC-NPs	Reference
					Fluorescence imaging system	Time (post- injection)	Control		
Skin tissue	Lung carcinoma (LLC)	C57BL/6 mice	Ce6-CdSe/ZnS QD	S.c., 1E6	UVP iBox Scientia	24 h	Naked Ce6-QD	>24 h	101
N/A	Lung carcinoma (LLC)	BALB/c nude mice	Ce6-MnO2 NP	I.v., 1E6	Bruker	24 h	Post-injection mice	>24 h	100
Bone marrow	Breast cancer (4T1)	BALB/c mice	Ce6@MSV NP	I.v., 1E6	IVIS	72 h	Unlabeled MSCs	>72 h	102
Adipose tissue	Lung carcinoma (A549)	BALB/c mice	Bi2Se3 NP	I.t., N/A	IVIS	24 h	Naked Bi2Se3 NPs	N/A	104
Bone marrow	Colon cancer (CT26)	BALB/c mice	Gd(III)-chelate NP	I.v., 1E6	12-bit CCD camera	2 h	Unlabeled MSCs	>2 h	72
Umbilical cord	Melanoma (B16F10)	C57BL/6 mice	Mn(II) and Gd(III) co-doped CuInS2-ZnS NP	I.v., 1E6	IVIS	6 h	PBS	>6 h	91
Bone marrow	Lung cancer (A549)	SCID Beige mice	PTX-PLGA NP	I.v., 2.5E5	IVIS	8 d	Unlabeled MSCs	>8 d	108

Abbreviations: I.t.=Intratumoral; I.v.=Intravenous; MSC=Mesenchymal stem cell; MSV=Multistage silicon vector; N/A=Not available; NP=Nanoparticle; S.c.=Subcutaneous; PLGA=Poly lactide-co-glycolic acid; PTX=Paclitaxel; QD=Quantum dot

**Table 5:** Nanotheranostic studies that have used PTT and PDT as therapeutic modality.

MSC origin	Cancer type (cell line)	Animal model	Imaging modality	Injection route and n of labeled cells	PTT agent	PTT paradigm			Temperature difference (C) for MSC-NPs	Temperature difference (C) for naked NPs	Tumor volume change for MSC-NPs	Tumor volume change for naked NPs	Reference
						Laser condition	Time (post- injection)	Control					
Umbilical cord	Prostate (PC-3)	Nude mice	PAI	I.t., 1E5	AuNS (anisotropic)	808 nm laser, 1.5 W/cm^2^, 10 m	3 d	Naked AuNS	10.9	8.1	200%	250%	70
Umbilical cord	Negative breast cancer (MDA-MB-231)	BALB/c athymic nude mice	PAI	I.t., 1E5	LDGI	808 nm, 1.5 W/cm^2^, 10 m	3 d	Naked LDGI	23	10	0%	600%	69
N/A	Breast cancer (MCF-7)	BALB/c nude mice	PAI	I.t., 1E6	AuNR@HPMOs-PTX	808 nm, 1.3 W/cm^2^, 5 m	24 h	Naked AuNR@HPMOs-PTX	16	16	150%	160%	98
N/A	Lung carcinoma (LLC)	BALB/c nude mice	Fluorescence	I.v., 1E6	Ce6-MnO2 NP	633 nm, 0.6 W/cm^2^, 5 m	24 h	Naked Ce6-MnO2 NP	N/A	N/A	0%	500%	100
Bone marrow	Breast cancer (4T1)	BALB/c mice	Fluorescence	I.v., 1E6	Ce6@MSV NP	405 nm laser, 100 mW, 15 m	0 h	Unlabeled MSCs	N/A	N/A	120%	150%	102

Abbreviations: AuNR@HPMOs-PTX=Gold nanorod hollow periodic mesoporous organosilica nanospheres with paclitaxel; AuNS=Gold nanostar; I.t.=Intratumoral; I.v.=Intravenous; LDGI=Lipid-Doxorubicin-Gold-Iron oxide nanocluster; MSC=Mesenchymal stem cell; MSV=Multistage silicon vector; N/A=Not available; NP=Nanoparticle; PAI=Photoacoustic imaging; PDT=Photodynamic therapy; PTT=Photothermal therapy.

**Table 6:** Nanotheranostic MSC studies that have used chemotherapy as therapeutic modality.

MSC origin	Cancer type (cell line)	Animal model	Imaging modality	Injection route and n of labeled cells	Chemotherapy agent	Chemotherapeutic regimen		Cumulative survival days for MSC-NPs	Cumulative survival days for naked NPs	Tumor volume change for MSC-NPs	Tumor volume change for naked NPs	Reference
						Dose	Control					
Adipose tissue	Brain astrocytoma (ALTS1C1)	C57BL/6JNarl mice	N/A	I.v., N/A	PLGA-PTX-SPIO NP	PTX dose of 2.5 mg/kg	Naked NP	35 d	25 d	N/A	N/A	115
N/A	Lung carcinoma (A549); ovarian cancer (MA148)	Athymic nude mice	Fluorescence	I.v., 5E5	PTX-PLGA NP	PTX dose of 0.2 mg	Naked NP	>70 d	55 d	N/A	N/A	71
N/A	Breast cancer (MCF-7)	BALB/c nude mice	PAI	I.t., 1E6	AuNR@HPMOs-PTX	N/A	Naked AuNR@HPMOs-PTX	N/A	N/A	150%	160%	98
Bone marrow	Lung cancer (A549)	SCID Beige mice	Fluorescence	I.v., 2.5E5	PTX-PLGA NP	PTX dose of 5 ug	PTX	160 d	110 d	0%	0%	108

Abbreviations: AuNR@HPMOs-PTX=Gold nanorod hollow periodic mesoporous organosilica nanospheres with paclitaxel; I.t.=Intratumoral; I.v.=Intravenous; MSC=Mesenchymal stem cell; N/A=Not available; NP=Nanoparticle; PLGA=Poly lactide-co-glycolic acid; PTX=Paclitaxel.

**Table 7:** Nanotheranostic studies that have used gene therapy as therapeutic modality.

MSC origin	Cancer type (cell line)	Animal model	Imaging modality	Injection route and n of labeled cells	Gene therapy agent	Transfection method	Gene therapy paradigm		Cumulative survival days for MSC-NPs	Tumor volume change for MSC-NPs	Tumor volume size for gene therapy control	Reference
							Dose	Control				
Placental	Glioma (C6)	Sprague-Dawley rats	N/A	I.v., 3E5	HSV-tk/GCV	MFION	100 mg/kg GCV solution	GCV solution	14 d	2%	17%	116
Adipose tissue	Ovarian (A2780)	Athymic nu/nu mice	N/A	I.p., 5E5	TRAIL	ZnFe2O4 MNP-silica-PEI/TRAIL	5 mg/kg recombinant TRAIL	Recombinant TRAIL	N/A	50%	100%	117
Bone marrow	Glioblastoma (U87 MG)	BALB/c nude mice	PAI	I.t., 1E5	TRAIL	Luminescence nanocomposite/TRAIL	N/A	Unlabeled MSCs	N/A	500%	N/A	103
N/A	Glioblastoma (U87MG)	BALB/cAnN.Cg-Foxn1nu/CrlNarl nude mice	MRI	I.c., 2E5	TRAIL	MTN-SPIO/TRAIL	N/A	Unlabeled MSCs	52 d	60-fold, 60,000%	N/A	89

Abbreviations: HSV-tk/GCV=Herpes simplex virus thymidine kinase/ganciclovir; I.c.= Intracranial; I.p.=Intraperitoneal; I.t.=Intratumoral; I.v.=Intravenous; MFION= Magnetosome-like ferrimagnetic iron oxide nanochain; MNP=Magnetic nanoparticle; MRI=Magnetic resonance imaging; MSC=Mesenchymal stem cell; MTN=Magnetic ternary nanohybrid; N/A=Not available; NP=Nanoparticle; PAI=Photoacoustic imaging; PEI=Polyethyleneimine; SPIO=Superparamagnetic iron oxide; TRAIL=TNF-related apoptosis-inducing ligand.
